# For autistic persons by autistic persons: Acceptability of a structured peer support service according to key stakeholders

**DOI:** 10.1111/hex.13680

**Published:** 2022-11-29

**Authors:** Alena Valderrama, Alejandra Martinez, Kathleen Charlebois, Lucila Guerrero, Baudouin Forgeot d'Arc

**Affiliations:** ^1^ Department of Social and Preventive Medicine, School of Public Health Université de Montréal Montréal Quebec Canada; ^2^ Research Center of the Sainte‐Justine University Hospital Montréal Quebec Canada; ^3^ Faculty of Medicine and Health Sciences McGill University Montréal Quebec Canada; ^4^ Centre Intégré Universitaire de Santé et Services Sociaux de Nord‐de‐l'Île‐de‐Montréal Montréal Quebec Canada; ^5^ Département de Psychiatrie Université de Montréal Montréal Quebec Canada

**Keywords:** acceptability assessment, mental health, mental health promotion, peer support, social support

## Abstract

**Introduction:**

Social support is a protective factor in the mental health of autistic people. Furthermore, prejudice regarding autistic people is a constraint for the development of social support programmes by autistic peers.

**Methods:**

The objective of this study is to describe the anticipated acceptability of structured peer support programmes for and by autistic persons. Fifteen key stakeholders (six autistic adults, four caregivers and five service providers) participated in in‐depth semistructured interviews. A qualitative thematic analysis of the content of the verbatim was carried out.

**Findings:**

We found that while a structured peer social support programme is acceptable to autistic people and caregivers, there was no consensus among service providers. The latter expressed doubts about the ability of autistic people to offer support. The framing of discussions between peers, the training of peer helpers, the support for autistic leadership and an organization that considers the communicational and sensory characteristics of autistic persons, could influence adherence to such a programme. Moreover, a space without service providers is an important condition for the acceptability of a peer support programme.

**Conclusion:**

A structured peer support service for and by autistic persons could be an innovative way to answer the unmet support needs of autistic people. It seems essential to anticipate potential barriers and facilitators and to communicate among health professionals to promote this approach and reduce possible prejudice about the ability of autistic people to offer support to their peers. More studies are necessary.

**Patient or Public Contribution:**

Fifteen key stakeholders who are involved in autistic people's trajectory of service and support participated in this research. We are a research team composed of healthcare professionals and researchers, in addition to one member of our team being an autistic advocate and a mental health peer‐support mentor. Two members of our team are also parents of autistic children. The comprehensibility of the questions for the interview was consulted and discussed with one autistic advocate‐collaborator.

## 1 INTRODUCTION

Approximately 1 in 66 children and youth are diagnosed as autistic in Canada.^1^ Autistic people are at high risk of having a low quality of life^2^ and of undergoing negative social experiences, health problems and mental health issues during their life course.^3^, ^4^ Whereas social participation at work and during leisure time along with the quality of social support networks are important protective factors,^2^, ^5^, ^6^, ^7^, ^8^ their needs in that regard remain unmet.^9^ Despite all this, to the best of our knowledge, there are no services with the primary objective of providing social support to autistic people.

### 1.1 What is social support?

Widely recognized as an important determinant of health,^10^, ^11^ social support is a process of social interaction that increases coping strategies, self‐esteem, sense of belonging and competence through the real or predictable exchange of practical or psychosocial resources.^12^, ^13^, ^14^ Indeed, social support is a mediator between stressful events and health. Supported persons would have more positive perceptions of their environment such as the belief that others can and will provide the necessary resources to help them and the perception of their ability to cope with various consequences of stressful events.^15^ Social support can be broken down into four main dimensions: emotional, appraisal, instrumental and informational. Specifically, emotional support involves receiving feelings of reassurance, protection or comfort. Appraisal support is about reassuring a person about their skills and values. Instrumental support is tangible aid, either material goods or services that directly assist people in need. Finally, informational support refers to the sharing of information and advice for solving problems.^12^, ^15^

### 1.2 Social support can be deployed by peers

In fact, social support can be deployed through formal (e.g., health professionals), semiformal (e.g., community organizations) and informal networks (e.g., families and friends).^15^ In particular, social support provided by peers with a common lived experience is known as peer support. The latter strategy has been proven in several chronic conditions to improve health outcomes and adherence to treatment and to reduce healthcare costs.^16^, ^17^ Efforts to integrate peer helpers into the healthcare system are underway in several areas.^18^, ^19^ In autism, peer support interventions have been proposed in the school setting as a way to promote the development of social skills and academic engagement, rather than for the benefit of social support as such.^20^, ^21^, ^22^, ^23^ These programmes, mainly consist of neurotypical students offering support to autistic persons, rather than autistic persons supporting autistic persons.

### 1.3 Social support by and for autistic people

Many autistic people appreciate and demand opportunities for socialization among autistic people and exchange in an autistic space of acceptance.^24^, ^25^ In research, there are promising experiences concerning autistic peer support.^26^, ^27^, ^28^, ^29^, ^30^ They suggest that a safe, structured space in a positive and welcoming environment would allow autistic people to feel accepted and understood (emotional support)^26^; to socialize around shared interests, and to recognize themselves in others (socialization and belonging support),^29^ while allowing for a better understanding of what autism is and developing coping strategies (informational and instrumental support) and a more positive perception of autism.^26^

These promising experiences are adolescent peer support groups of Weidle et al.,^30^ mentoring dyads of Martin et al.,^29^ and support groups for newly diagnosed adults of Crane et al.^26^ The purpose of the autistic adolescent support groups was to provide a space for the development of autistic peer relationships in a hospital setting. Mentoring dyads aimed to improve the well‐being of mentees in a school setting. The adult support groups were designed to evaluate the benefits of an autism training tool offered following diagnosis in a community setting. In each of these programmes, participants carried out a qualitative evaluation of satisfaction and an evaluation of the benefits received, and their feedback was positive overall. Even more, Hotez et al. co‐developed a programme with autistic people to support students to transition into postsecondary education. In this programme, two autistic people successfully acted as mentors.^28^ Finally, Gillespie‐Lynch et al.^27^ co‐developed another mentorship programme for autistic students in postsecondary education where autistic students acted also as mentors. In fact, autistic people, possibly due to their particularities in terms of communication and social cognition, often experience difficulties in establishing informal relationships of social support.^31^ Even more, myths and prejudices about autistic people such as lack of empathy and interest in social relations^32^ might hinder the establishment of social support services for and by autistic peers among services providers or services users like families. That is why, from a perspective to evaluate and develop complex interventions,^33^ we wanted to study the acceptability of a structured support programme for and by autistic peers in the Quebec context.

### 1.4 Acceptability assessments for the development of a structured peer support service

Acceptability can be and should be assessed before engaging in an intervention. We define a structured programme as an organized set of activities and services carried out simultaneously or successively with dedicated resources to achieve specific objectives in relation to specific health problems, in a defined population.^34^ Therefore, the objective of this research is to describe the anticipated perception of the acceptability of such a programme, from the perspective of key stakeholders like autistic persons, caregivers or social workers who are involved in autistic people's trajectory of service and support.

Sekhon et al.^35^ define acceptability as the extent to which people delivering or receiving a healthcare intervention consider it to be appropriate, based on their anticipated or experienced cognitive and emotional responses to the intervention.^35^ For the purposes of this study, acceptability is defined in terms of what is required for a structured social support programme provided by autistic peers for it to be considered a source of support for autistic people. Several factors can influence participants' perceptions of acceptability before participating in the intervention. Therefore, we used the theoretical framework of acceptability (TFA) which consists of seven component constructs: *Affective attitude* towards the intervention. *Ethicality* or the extent to which the intervention has a good fit with an individual's value system. *Intervention coherence* or the extent to which the participant understands the intervention, and how the intervention works. *Perceived effectiveness* or the extent to which the intervention is perceived as likely to achieve its purpose. *Self‐efficacy* or the participant's ‘confidence that they can perform the behaviour(s) required to participate in the intervention’. *Opportunity costs* or the extent to which benefits, profits or values must be renounced to engage in an intervention which influences adherence and participation. *Burden* or the perceived amount of effort that is required to participate in the intervention and reasons for discontinuation.^35^

## 2 METHODS

### 2.1 Study design

A qualitative descriptive design^36^, ^37^, ^38^ underpinned this study with the aim of ascertaining perceptions as to the acceptability of structured peer social support for and by autistic persons by key stakeholders. We are a research team composed of healthcare professionals in public health, service organization, mental health and qualitative research, in addition to one member of our team being an autistic person and a mental health peer‐support mentor. Two members of our team are also parents of autistic children.

Since our focus was on life experiences and subjective perceptions, the use of qualitative methodology was appropriate.^39^, ^40^ In‐depth semistructured interviews with key stakeholders were carried out. Participants did not receive incentives for participation. Table 1 shows only some characteristics of the participants (key stakeholders) to preserve their anonymity.

**Table 1:** Characteristics of the participants

Key stakeholders	Main characteristics
Seven autistic adults (AA)	Six females and one male, who communicate orally, four having received a diagnosis in the last 2 years.
Four caregivers (CV)	All females, two of the children were nonverbal, three children lived with their caregivers, three of the children were adults and one was an adolescent.
Four service providers (SP), it means health professionals or social workers	All females, two persons of community organizations that offer support to autistic people and three psychoeducators from the health system.
Total of 15 key stakeholders	

### 2.2 Sampling and recruitment of participants

Participants were recruited using a purposive criterion sampling strategy.^41^ Potential participants included (1) people that self‐identified as autistic people (AA), (2) caregivers of autistic persons (CV) and (3) service providers (SP) such as health professionals or social workers. More specifically, service providers were required to have at least 5 years of experience working with autistic people (adolescents or adults) in the health and social services network in Quebec to be eligible to participate. Moreover, the participants should be living in Quebec and speaking fluently in French. In addition, email invitations were sent to two autistic people, three health professionals and two community organizations known for their involvement in supporting autistic people. All of them accepted to participate in an interview except one autistic person who did not respond to the invitation. Thus, eight people were recruited by invitation and seven more through CHU Sainte‐Justine mother and child Hospital centre social media platforms (six AA and two CV). These people were considered key stakeholders because they have valuable information, as health professionals, as people directly concerned or as caregivers involved in their community. The cases chosen should provide the greatest possible wealth of information for an in‐depth study of the research question.^42^ A. M. carried out the recruitment in June and July 2020. We conducted data analysis in parallel with ongoing data collection. We stopped at 15 interviews. The sample size was not predetermined and was based on the richness of the data. This is because we were able to answer the research questions.

### 2.3 Data collection

Fifteen in‐depth semistructured interviews with these key stakeholders were carried out. After receiving training in qualitative research, A. M. conducted the interviews in July and August 2020. While most interviews were conducted via Zoom, one was by phone. One autistic person was asked to answer the questions in writing, and another person also added answers in writing after the interview. Interviews were conducted in French and lasted approximately 1 h. Finally, the interviews (by Zoom and by telephone) were recorded and transcribed by A. M.

We developed an interview guide based on the TFA and adapted it to each group. Autistic persons were asked about their needs surrounding the four types of social support: emotional, appraisal, instrumental and informational,^12^ to understand their attempts to respond to those needs. An example of a question was, *when you are going through a difficult situation or you are going through negative emotions, what do you do to get better? Has this happened to you lately? How did that happen?* We also wanted to examine the role of peer support in the course of their efforts to respond to their needs in terms of support.

The second topic centred on how a peer support service would be received. An example of a question was, *could the life experiences of another autistic person be a reference, a model, an inspiration or a source of support that could help you better face problems if you have them? If yes, why? If not, why?* Based on research experiences of modalities of peer support, we also explored the participants' perception of three possible forms of social support for peers: support groups, mentors and social support online.^26^, ^27^, ^28^, ^29^, ^30^ An example of a question was, *what would be the challenge to overcome in a possible service?* The comprehensibility of the questions was consulted and discussed with one autistic person collaborator. Changes were made afterwards. While different interview guides were used for caregivers and service providers/healthcare professionals, respectively, both interview guides comprised the following topics: (1) what they consider to be autistic persons' social support needs and (2) the acceptability of a structured programme. The interview guides were developed in French (available alongside data in Dataverse).

### 2.4 Data analysis

A qualitative thematic analysis^43^ of the content of the verbatim was carried out by three researchers (A. M., A. V. and K. C.) using the NVivo 12 software according to the TFA.^35^ The purpose of the thematic analysis was to identify the semantic units that constitute the discursive universe of the statement to reformulate the content in a condensed form.^43^ The analysis was carried out in four stages: (1) the identification of significant ideas, (2) their categorization, (3) within each group of targeted actors (intragroup analysis) and (4) between the groups of targeted actors (analysis intergroups).

To enhance the trustworthiness of the data analysis we went through the next stages.^44^ A number has been provided to each participant to maintain anonymity. Then, the first author reads the data several times to repair emerging issues, trends and identify patterns among coded categories. Therefore, we developed an initial codebook based on these initial readings of the interview data, along with aspects of the theoretical framework. The first coding was carried out by A. M. A. V. performed 60% of the encodings. Double‐coded interviews were compared, and disagreements were discussed until consensus was achieved. So, interview data were thus initially coded, based on: (1) the needs of autistic people in terms of social support (i.e., emotional support, informational support, appraisal and instrumental support),^12^ (2) the past experiences of social peer support: positive experiences and drawbacks, (3) the acceptability of a structured service of social peer support for and by autistic persons (more precisely, around the seven constructs of the TFA: affective attitude, burden, perceived effectiveness, ethicality, intervention coherence, opportunity costs and self‐efficacy)^35^ and (4) the possible barriers and facilitators of a peer support service.

Afterwards, for the purposes of theme development and refinement, a template or framework was then developed following adjustments stemming from discussion among all the researchers involved in the study, until consensus was reached.^45^, ^46^ Theme development and refinement were characterized by an iterative process in which interview data and theoretical constructs are confronted and contrasted.^47^ Ambiguous verbatim were also discussed as a team and reclassified. Then, K. C. performed a third data analysis. Finally, results were presented to the rest of the team and further discussed until a consensus was reached.

## 3 FINDINGS

Theme development was guided by concepts stemming from the Acceptability framework (TFA) along with concepts pertaining to various types of social support.^12^ Table 2 shows how the framework was adapted for the data analyses (Table 2). Findings point to four major themes: (1) Social support needs and what peer support might mean; (2) The underpinnings of a structured social support programme provided by peers: ethicality and perceived effectiveness; (3) Practical considerations for implementing a structured peer social support programme: self‐efficacy and intervention coherence and (4) The costs and potential barriers around the development of such a service: burden and opportunity costs.

**Table 2:** Acceptability framework (Sekhon et al.) adapted to issues around social support provided by autistic persons

Concept	Definition	Issues pertaining to social support provided by autistic people
Affective attitude	Feelings about an intervention (social support programme)	Interest in or resistance towards social support given by autistic peers Understandings of social support and social support needs Perceived social support needs (emotional, informational, appraisal and instrumental support) Recognition around the need for social support Gaps in social support
Ethicality	Fit between intervention and individual's value system	Sharing and understanding of similar experiences with other autistic peers
Intervention coherence	Understanding of the intervention by the individual and how it works; fit between components of intervention and intended aim	Understandings of the intervention; ways of defining what the intervention does Potential and limits of social support by autistic peers Comparisons with professional services Views (preconceived notions) around ability of autistic people to understand intervention among clinicians and healthcare professionals Partnerships and gaining credibility in the community
Perceived effectiveness	Perception of likelihood of intervention to achieve its purpose	Possibility to engage in mutual understanding Flexibility in meeting different social support needs Role of peer supporters Role of and support from healthcare professionals around social support by autistic peers
Self‐efficacy	Participant's confidence in taking part in the intervention	Frequency of intervention Routine of intervention Format of meetings (e.g., videoconferencing) Type of discussions
Opportunity costs	Benefits, profits and values given to participate in the intervention; what has to be or was given up to engage in the intervention	Potential barriers to participating in and implementing the intervention Need for training
Burden	Perceived amount of effort required to participate in the intervention	Pressure to share experiences Compatibility among autistic persons taking part in social support intervention Extent to which the intervention is adapted to sensory characteristics of autistic peers Extent to which the intervention is adapted to communication characteristics of autistic peers

### 3.1 Social support needs and what peer support might mean

Underlying perspectives surrounding the acceptability of structured social support programmes by and for autistic people were participants' experiences seeking social support, the lack of social support in their daily lives as well as the gaps in terms of social support needs for autistic people.

#### 3.1.1 Seeking social support and sometimes finding it

First of all, the need for social support was recognized by participants in this study, as reflected in this comment by an autistic person:

Of course, one thing that is common to everyone is that we all have a need to talk about what we are going through personally, regardless of the level of difficulty … Even if things are going well, it is certainly relevant to express our daily concerns. (AA5)

Autistic participants mentioned encountering issues with instrumental support (material and financial resources). More specifically, they experienced challenges accessing services access, for example, to transportation, something that was echoed by caregivers and healthcare professionals. Also, the need for informational support was raised by autistic persons as well as parents/caregivers who took part in this study. Some autistic participants mentioned the internet as a source of information as well as documentation found through the healthcare system. Among the information that was sought, one autistic person mentioned the need for information on how to manage one's emotions, food sensitivity and sensory perceptions. Information needs pertaining to finances, work, education, love, sexuality as well as identity were raised by caregivers interviewed in this study.

Two autistic persons mentioned being partially successful in meeting their support needs, saying that they were counting on someone in the family. Their mothers primarily were the source of emotional and instrumental support. However, for two service providers, the need for social support could be met rather through social integration, especially at school or at work, but also through rehabilitation services. Therefore, the perspective of the social support needs of autistic persons was not the same, whether it was the people concerned or the other key informants.

Service providers and caregivers interviewed in this study tended to focus on the need for socialization and noted the loneliness experienced by the autistic people they encountered. Thus, socialization groups with other autistic persons, facilitated by professionals and social media groups such as Facebook were the sources of socialization outlined by some autistic persons. Above all, unmet needs for social support were mentioned among autistic people interviewed, as reflected by one of the participants:

So I don't feel support, I don't feel anything useful. I don't feel an understanding or anything. So I can say very clearly that I don't feel any support. (AA2)

#### 3.1.2 How an autistic peer might respond to social support needs

One caregiver considered providing informational support as central to the role of an autistic peer support worker. Another mother considered them (peer support worker) to be best equipped to help autistic persons compose with a neurotypical society in a pragmatic way.

With regard to emotional support, autistic participants expressed the need to find the ‘ideal’ autistic peer support worker (as put by one autistic person interviewed for this study), that is someone who can understand their feelings from the perspective of an autistic person, as reflected by one mother interviewed in this study:

If it could be someone who can explain well according to his perception of the environment […] I think that would be a win for him [hers son]. (CV4)

Healthcare professionals also mentioned the role autistic peer support workers could play in providing emotional support, with caregivers acknowledging the need for emotional support to prevent psychological distress. Alongside emotional support was the need to develop autistic persons' self‐esteem. Autistic peer support worker was deemed suitable to provide such support, particularly according to the healthcare professionals interviewed for this study.

Finally, stigmatization remained an issue according to the autistic people and caregivers who took part in this study, hindering social support as well as the self‐esteem of autistic persons. Autistic participants mentioned feeling judged on account of their autistic particularities. Further aggravating the situation was the categorization of autism along with false beliefs around the condition.

Autistic adults and caregivers interviewed indicated that such a structured social support service would be accepted, as this verbatim shows:

A peer support service, I would certainly make good use of it. I could even tell you that I am in contact with adults in my condition, and we help each other a lot…. (AA5)

Peer support was deemed relevant for autistic people as well as for parents. Peer support was underlined by one parent as a form of social support for autistic persons:

I think there is nothing better than one person seeking to understand another person with the same condition. (CV4)

All autistic people and caregivers had a positive attitude towards structured peer social support services. Such a service was deemed compatible with their values of mutual aid. Participants understood that peer support might be possible due to the life experience of peers and their ability to share it. Table 3 shows verbatim according to the perceptions of the participants and the different types of support plus their affective perception towards this service (Table 3).

**Table 3:** Social support needs and what peer support might mean

*Social support needs/verbatims*	
Seeking social support and sometimes finding it.	
Emotional support	‘I cry a lot, I call in different places but I have a feeling that people don't really understand what autism is. Just to make yourself understood creates conflict’ (AA1).
	‘The only person who understands me is my son because he is also atypical’ (AA2).
	‘I read on forums … I am on Facebook groups where I chat. Recently, for example, I had a big concern to share … so uh, I wanted to share that in order to evacuate’ (AA5).
Instrumental support	‘I call on people, generally strangers, for a service’ (AA4).
	‘I have no one to help me in an emergency’ (AA6).
Appraisal support	‘I have a feeling that for a part of society, no matter how good or successful I am at something, I am the “incapable autistic”’ (AA6).
Informational support	‘My social worker. Otherwise, I found it in books or on websites. I also have an autistic friend who has read everything and who gives me good summaries with all the details’ (AA4).
*Social support needs/verbatims*	
The perspective of the social support needs of autistic persons was not the same, whether it was the people concerned or the other key informants.	
Social support	‘It is against their nature to be in a social environment. The best support for a young adult with autism is individual support from a professional, whether an educator or a social worker or a psychologist’ (SP4).
*Affective attitude to a peer support service/verbatims*	
An autistic peer might respond to social support need of other autistic persons.	
	Autistic person: ‘Even I think about it. Maybe later becoming a peer helper, because I have a certain lived experience and I can give to others. I think it's more through experience that we can share, so that things get better with people. Instigate light in them’ (AA1).
	Family member: ‘I think there is nothing better than one person seeking to understand another person with the same condition’ (CV4).
	Healthcare professional: ‘I think it's a great way to provide quick and effective support for people with peers who experience much the same reality’ (SP01).

### 3.2 The underpinnings of a structured social support programme provided by autistic peers: Ethicality and perceived effectiveness

Past experiences with social support initiatives underpinned participants' concerns around the ethicality and effectiveness of a structured social support programme. More specifically, experiences among some of the autistic persons interviewed with unstructured spaces (whether through the internet or community organizations) led them to emphasize the possibility to share their experiences in the absence of healthcare professionals. They also underlined the need for guidance and training among those moderating a support group. Table 4 outlines autistic persons' perspectives based on the requirements of social support initiatives stemming from their past experiences, which centred on the possibility to share one's concerns and experiences freely. This not only spoke to the ethicality of social support initiatives but also their effectiveness.

**Table 4:** What should underpin peer social support: Ethicality, perceive effectiveness and past experiences with unstructured spaces

	Verbatim
Ethicality	‘I participate in a social group; we exhibit what we are experiencing and then everyone can give a comment. We meet between us and then if someone has exposed a situation, we can validate saying yes. I experienced a situation like that or not, then we come to understand each other’ (AA4).
	‘I like my socialization group because we can talk about whatever topics we want, there are no service providers’ (AA5).
	‘People who have gone through adversity who are able to share their life experience … Without even giving advice that could indeed give me ideas’ (AA2).
Perceived effectiveness and past experiences with unstructured spaces	‘It does me a lot of good to have friends who are autistic like me. It's better than therapy. At least I can have a social network’ (AA2).
	‘We understand each other, we don't need to look each other in the eyes to talk to each other, we won't use all of our efforts like when talking to a non‐autistic person’ (AA4).
	‘The difficulties in terms of theory of mind, in terms of reading social issues, or in terms of cognitive flexibility precisely make them more vulnerable in terms of mentoring attitude so it's not just anyone can be a mentor and the person must be trained and at the limit supervised or coached’ (SP1).

Furthermore, the need for a structured social support programme to be adaptable and flexible to individual needs were the focus among some autistic participants. Among the autistic persons interviewed, the effectiveness of such a service resided in the possibility for autistic people to engage in mutual understanding of their experiences. Autistic participants shared a similar view of the role of peer helpers as primarily a source of comfort, informational and instrumental support, in addition to encompassing a socialization role. For service providers, peer helpers represented rather a functional resource to health services, as underlined by one service provider:

The peer helpers could certainly play a role of reception, validation and comfort. At best, it might play a role of a bit of guidance, to the right services, the right resources. (SP5)

With the exception of some service providers, effectiveness and ethics went hand in hand when it came to peer‐to‐peer social support programmes (Table 4).

### 3.3 Practical considerations for implementing a structured peer social support programme: Self‐efficacy and intervention coherence

Practical considerations over the implementation of a structured peer social support programme entailed both the possibility for autistic people to participate as well as the need to disseminate such a programme within the wider community. Of particular concern was the location. In general, autistic persons reported that the location of the service was irrelevant, provided it is easy to access and sensory perceptions are considered. An autistic participant and a service provider mentioned videoconferencing as an option, particularly within the context of the COVID‐19 pandemic and even after. Some parents made a similar suggestion, with one mother mentioning that it made little difference to her whether the support programme occurred in a hospital, a clinic or a community organization:

[The autistic person] must be able to identify to the group […], but for the rest, I honestly think that it would not matter if [the service] was in a hospital, a clinic or a community organization. I think that is a detail. (CV4)

Compared to the autistic participants, healthcare professionals interviewed in this study expressed more concern over the location of the support programme, preferring it to take place within a local government health agency (centre local de services communautaire [CLSC]). The latter was thought to ensure the accessibility and continuity of such a programme as a service. Nonetheless, a community organization was also deemed acceptable. However, healthcare professionals interviewed for this study remained divided with regard to a support programme being situated within a hospital, for fear that the programme would be perceived as a medical service by autistic persons:

It's all in the art of presenting the service not as … a health service, but as a support service. (SP01)

Some autistic people interviewed prioritized a space without health professionals, supervised discussions and support for autistic leadership as factors that would influence their adherence to a potential social support programme (Table 5). However, there was a lack of consensus among the service provider participants. Some expressed doubts about the ability of autistic people to offer support to others. In addition, regarding the component *intervention coherence* or the extent to which the participant understands the intervention, and how the intervention works, some suggested that such a programme would be a low‐quality health service.

**Table 5:** What a structured peer social support programme requires: Practical considerations

Intervention coherence	
Dissemination and promotion of the support programme	‘The dissemination and promotion on the internet through our own services, to integrate it to our service and inform schools that it is a service we are offering’ (SP01).
	‘Community organizations, promotion through social media […] promote it to parents by presenting the project. I also think it could be in the school system and in the workplace where there are autistic people’ (SP02).
	‘Promote it in hospitals where psychiatrists receive adolescents or adults’ (PS02).
	‘Maybe it could be done through the meeting with the doctor, so […] he would have a presentation I think during that meeting, well … The person would already be less afraid I think of seeing this person again then it would give credibility to the person if they were presented by the doctor as being a really useful service and that they could therefore benefit from using it’ (CV4).
Comparison with professional service	‘The service offered by the peer helper will not be able to go as far as a professional service. It will not be able to give the same depth’ (SP1).
Self‐efficacy	
A framework for discussion	‘A lot of people talk a lot about their difficulties, it's good to hear it once, there is compassion … we offer compassion because the idea is also to empty your bag, but then I would like us to move on to something else’ (AA2).
Support to autistic leadership	‘She tried to intervene the time I said in the group of friends that I was afraid of catching Covid‐19 by picking up my recycling bin and she told me that she finds my overreaction. Afterwards, Madam leads a support group without having experience in intervention. I felt judged’ (AA4).
Predictable	‘Once a week is good [.] because I make an appointment. So, I'm going to put it in my routine and then I have no problem [.]’ (AA2).
	‘A pleasant, quiet room, no matter where’ (AA6).
A space without service providers	‘For example, we meet with friends, then we just talk to each other, without there being a predefined topic. Probably a trained person, who knows autism, who does not judge, who does not act by intervening, that is to say who does not give orders or tools, for example’ (AA4).

Other than the location was the frequency of the social support meetings, with emphasis on a fixed routine and a regular schedule, particularly among autistic people interviewed for this study as well as parents. Structured meetings with clear objectives and themes were also considered important in the design of a social support programme for autistic people, both by healthcare professionals and autistic participants. In addition, for one autistic person, a mentor available in times of crisis was noted:

Once a week is good […] because it is a fixed appointment. I then have a routine and I don't have any problems […], but it would also be [good to have a mentor if I go through a crisis for example. (PA02)

Concerns over how best to promote or disseminate a structured peer support programme in the community were raised among healthcare professionals interviewed for this study. They expressed some reluctance regarding the benefit of a peer support programme considering it often as improvised. Underlying such concerns was the need to recruit potential autistic peer support workers.

The importance of forging partnerships with other social and health organizations as well as the school system was emphasized by several healthcare professionals interviewed, namely as a way for such a service to gain credibility. This was thought of as carrying the potential to offset any reluctance around its implementation and ensure buy‐in around such a programme. The need to educate healthcare professionals and clinicians about peer support was considered another strategy to obtain buy‐in around such a programme.

Finally, a parent highlighted the benefit of the programme being presented by a doctor as a way to augment its credibility. One autistic person emphasized the need for support immediately following a diagnosis corroborating the relevance of having doctors suggest the programme to patients.

### 3.4 The costs and potential barriers around the development of such a service: Burden and opportunity costs

Regarding the burden and opportunity cost components of the TFA model, autistic persons reported untrained or skilled peer helpers and/or an organization that does not consider the communicational and sensory peculiarities of autistic people as reasons for dropping out of a social support programme. Among healthcare professionals interviewed for this study, training for potential autistic peer support workers was nonnegotiable. As to what this training would comprise, autistic participants emphasized the need for a peer support worker to know when to intervene and how, for example, when an autistic person experienced sensory overload. Parents also pointed out the need for autistic peer support workers to accompany the autistic person as well as to be knowledgeable of existing services and resources for autistic people. Healthcare professionals raised the latter point, along with the need to train autistic peer support workers about communication techniques, stigmatization issues as well as about autism in general.

Even with trained autistic peer support workers and a programme adapted to the communication and sensory peculiarities of autistic people, some autistic participants remained sceptical given issues around compatibility among autistic people taking part in such a programme. Adding to this scepticism was the pressure to share one's experiences with other members of the group. Finally, apart from possible issues accessing the service, autistic persons interviewed did not view any further difficulty using such a service. Table 6 shows the costs and potential barriers for a structured social support programme for and by autistic peers according to the participants.

**Table 6:** Possible barriers for a structured social support programme by autistic peers

Opportunity costs	
Training peer helpers	‘That depends on the people. I know a girl, when she tells her stories, she picks up bad comments, judgmental reports. That's why I said that intervention training for these peer helpers would be good’ (AA4).
	‘It has to be autistic qualified to listen and to put things into perspective. He must be able to transform what we are saying into a concrete tool for improvement. If we manage to progress, to develop tips, it has to be beneficial anyway to follow me on the long term. That interests me. So, it must be a qualified autistic, who has a qualification’ (AA2).
	‘The negative point I could say is maybe someone who doesn't have a lot of training to help you or it's someone who has a bigger problem’ (AA4).
Burden	
Adapting to communication particularities	‘A conference via an internet link will tire me out. My tolerance level is 30 minutes, but when I chat in writing I can chat for hours’ (AA2).
Adapting to sensory particularities	‘Because of the sensory perceptions, the light level, the temperature and all that stuff, I know these things are things that I couldn't stand, so it is not even worth thinking about going to a particular environment’ (AA1).
	‘A place that is easy to access and not too noisy and with nonaggressive lighting’ (AA4).
No obligation to share	‘It is as if in these groups it is obligatory to speak about our sufferings and not to speak about what we were proud of and what we accomplished’ (AA2).
Considering compatibility issues	‘With compatible people. Not all autistic people are compatible’ (AA5).
	‘The big disadvantage is falling into a relationship of convenience with the peer helper. For example, “I had a lot of difficulty finding a job.—Ah yes me too, uh I understand you but me it's worse”’ (SP1).

## 4 DISCUSSION

### 4.1 A structured peer support service for and by autistic persons is acceptable for autistic persons and caregivers and for some service providers

To our best knowledge, this research is the first to study the acceptability of a structured programme of social support for and by autistic peers from the perspective of different key stakeholders. The interviews of autistic people and caregivers carried out in this research showed their acceptance of a structured autism peer social support programmes.

They self‐identified with the values of solidarity of peer support and underlined the importance of the experiential knowledge of autistic peers. However, at the same time, some service providers expressed their doubts about the ability of autistic people to offer this support. Furthermore, the success conditions could be the training of peer helpers, the framing of discussions, the support for autistic leadership of the service and an organization that considers the communicational and sensory characteristics of autistic persons. Finally, a space without service providers was an important condition for the acceptability of a peer support programme.

There are many reasons why peer support for and by autistic persons could be effective. The Double Empathy Problem suggests a higher rapport during interactions between pairs of the same neurotype,^48^ indicating a smoother relationship between autistic people. Autistic people possess a distinct mode of social interaction style, rather than demonstrating social skills deficits.^49^, ^50^ In addition there is a greater affinity between them and a sense of belonging as well as some preference on their part for interaction with other autistic people.^51^, ^52^ Even more, information sharing between autistic people could be more effective than the information transfer between autistic and nonautistic people.^49^ Although the autistic participants in our study did not request a space without nonautistic people, they requested a space without health professionals (service providers), who are almost always nonautistic.

### 4.2 Possible barriers and facilitators for the organization of a structured peer support service for and by autistic persons

In our study, all autistic participants had experiences with spaces of spontaneous exchanges between autistic people. Our study shows that autistic people make use of unstructured spaces of peer support through social media, which is in agreement with Zhao et al.^53^ They express positive experiences, but also negative ones, for example, the lack of framing of discussions.^54^ Therefore, as reported by Martin et al.,^29^ training peer helpers and support to autistic leadership, could be a facilitator to improve the disadvantages of unstructured spaces through social media or others. Even more, as described by Cherba et al.^55^ the direct implication of health professionals in peer support services could be sensed as a manifestation of authority or of an unequal power relationship.

Our study also shows that a peer support service might encounter scepticism from service providers, related to the capacity of autistic persons to offer support to others. This might be related to the experience of participants with verbal communication skills of autistic persons, which is greatly heterogeneous. It could also be related to the implicit stigma of autistic people in health care,^56^, ^57^ and could underlie this lower acceptability. Further inhibiting the establishment of a peer support service is a misunderstanding of the social support needs of autistic people as well as insufficient knowledge of peer helpers' role on the part of service providers.^18^ Efforts should also be made to combat the stigma of autism in healthcare settings and to improve the acceptability of peer helper services in health care. Therefore, improving acceptability by health professionals is important to integrate a service like this across the continuum of healthcare services or into the community.

### 4.3 Next steps

There are further challenges in planning and organizing a structured programme of social peer support for and by autistic persons. There is a need of developing adequate training for peer helpers, of offering them the necessary mentoring and support for safe services, like resources for risk management, abilities for community management or access to communities of practice of autistic peer helpers.

Peer support workers in mental health are aimed at alleviating symptoms and risks associated with illness, disease control and well‐being with illness.^19^, ^58^ The autistic participants in this study did not perceive themselves as sick people. The kind of peer support asked for focuses mainly on concepts of health promotion in a vulnerable population. This is why we need to understand how a recovery approach can be complementary to strategies of health promotion for promoting positive mental health^59^ with social support to autistic persons by autistic peers.

### 4.4 Study limitations

The study highlights a gap between service providers and autistic participants regarding the perceived capacity of autistic persons to offer peer support. Some service providers might have been influenced by general stereotypes about autism (e.g., ‘It is against their nature to be in a social environment’), while autistic participants might have been referring to their own abilities. Or maybe autistic participants and service providers referred to different situations when talking about autism, due to the heterogeneity of autistic people receiving social or health services. We did not explore the particularities of autistic people to whom these workers offered services.

We have to admit that the meaning of social support for autistic people was not enough explored in our study. We used one of the most accepted definitions of social support.^12^ A more contextualized approach to social support would be necessary to improve research for useful interventions and practices in a particular context,^14^ like in autism.

In addition, while this study did not clarify how saturation was established, it was nonetheless possible to capture a certain level of wealth and depth of information based on the interviews that had been conducted, rendering the sample size appropriate for the purposes of this study.^60^, ^61^ We interviewed different groups of key stakeholders to account for diverse perspectives. We are aware of the fact that the sample is not representative of any of these groups. This is possible that the characteristics of this sample of autistic persons like being an adult, who was recruited by social media influenced their interest in using social support. We only interviewed autistic adults living in Quebec and speaking fluently in French who expressed themselves orally. Even if these results cannot be generalized to all autistic persons and stakeholders, this research gives the first insight into this important topic. Additional co‐construction work is needed to confirm our findings and assess our proposals.

## 5 CONCLUSION

A structured peer support service for and by autistic persons is acceptable for autistic persons and caregivers and for some service providers. To organize a structured autism peer social support programme, we assessed the acceptability of providers and recipients. The training of peer helpers to facilitate discussions among peers, an organization that considers communicational and sensory characteristics of autistic persons and support for autistic leadership of the programme appear as factors that would influence adherence to such a programme. In addition, a provider‐free space is an important condition for the acceptability of such a service. It seems essential to communicate among health professionals to promote this approach and reduce possible prejudice about the ability of autistic people to offer support to their peers. Finally, a structured peer support service for and by autistic persons could be an innovative way to answer the unmet support needs of autistic people. More studies are necessary.

## AUTHOR CONTRIBUTIONS

Alena Valderrama designed the study. Alejandra Martinez oversaw recruitment and data collection. Alena Valderrama, Kathleen Charlebois and Alejandra Martinez carried out the data analyses. All authors discussed the results and contributed to the article draft. All authors have reviewed and approved the article before submission. This article has been submitted solely to this journal and is not published, in press or submitted elsewhere.

## CONFLICT OF INTEREST

The authors declare no conflict of interest.

## ETHICS STATEMENT

The study obtained approval from the Research Ethics Board of the Sainte‐Justine University Health Center (CHU Sainte‐Justine) project 2020‐2858. Online informed consent was obtained from all the participants. All authors have reviewed and approved the article before submission. This article has been submitted solely to this journal and is not published, in press or submitted elsewhere.

## Data Availability

Alena Valderrama—Dataverse (Université de Montréal). Valderrama, Alena, 2022, ‘For Autistic Persons by Autistic Persons: Acceptability of a Structured Peer Support Service According to Key Stakeholders’, https://doi.org/10.5683/SP3/3L2FXU, Borealis.
